# Providing a fast conversion of total dose to biological effective dose (BED) for hybrid seed brachytherapy

**DOI:** 10.1120/jacmp.v13i5.3800

**Published:** 2012-09-06

**Authors:** Jakub Pritz, Kenneth M. Forster, Amarjit S. Saini, Matthew C. Biagioli, Geoffrey G. Zhang

**Affiliations:** ^1^ Radiation Oncology Moffitt Cancer Center Tampa FL; ^2^ Physics Department University of South Florida Tampa FL

**Keywords:** hybrid seeds, brachytherapy, optimization, BED

## Abstract

Optimization of permanent seed implant brachytherapy plans for treatment of prostate cancer should be based on biological effective dose (BED) distributions, since dose does not accurately represent biological effects between different types of sources. Currently, biological optimization for these plans is not feasible due to the amount of time necessary to calculate the BED distribution. This study provides a fast calculation method, based on the total dose, to calculate the BED distribution. Distributions of various numbers of hybrid seeds were used to calculate total dose distributions, as well as BED distributions. Hybrid seeds are a mixture of different isotopes (in this study ^125^I and ^103^Pd). Three ratios of hybrid seeds were investigated: 25/75, 50/50, and 75/25. The total dose and BED value from each voxel were coupled together to produce graphs of total dose vs. BED. Equations were then derived from these graphs. The study investigated four types of tissue: bladder, rectum, prostate, and other normal tissue. Equations were derived from the total dose – BED correspondence. Accuracy of conversion from total dose to BED was within 2 Gy; however, accuracy of conversion was found to be better for high total dose regions as compared to lower dose regions. The method introduced in this paper allows one to perform fast conversion of total dose to BED for brachytherapy using hybrid seeds, which makes the BED‐based plan optimization practical. The method defined here can be extended to other ratios, as well as other tissues that are affected by permanent seed implant brachytherapy (i.e., breast).

PACS number: 87.55.de

## I. INTRODUCTION

The linear‐quadratic model is currently the most used model that quantitatively describes the survivability of a cell line for a given amount of radiation. Deriving from the linear‐quadratic model, the biological effective dose (BED) describes the biological effects of radiation.^1^, ^2^ Since BED provides a way of including radiobiological parameters, quantitative treatment expectations can be obtained.^(3)^ It has been suggested that BED be used as a guide in clinical decision‐making.^3^, ^6^ This requires the calculation of BED from dose. Several papers have addressed the conversion of dose to BED for fractionated external‐beam radiation therapy,^1^, ^3^, ^6^ in brachytherapy,^5^, ^7^, ^8^ and for composite modalities (external beam with brachytherapy).^(9)^

With modern technologies, multiple radiotherapy treatment modalities are available to treat cancer. An example of such radiotherapy is prostate cancer being treated with external beam radiotherapy initially followed by seed implant brachytherapy as a boost treatment. Because of the dose rate differences between various treatment modalities, the radiation dose cannot be directly used to compare treatment outcomes of the various modalities. Just as in multimodality treatments, the radiation from hybrid seeds used will be delivered at different dose rates, since hybrid seeds have a mixture of isotopes. For a lesion treated with different dose rates, the total dose from the treatments is not meaningful. The biological effective dose (BED), instead of the total dose, from the different modalities or dose rates should be used for treatment plan evaluation.

In brachytherapy, BED‐based optimization would be preferred in planning when multiple dose rates are involved. Currently, dose‐based optimization is common, but BED‐based optimization is not available in commercial treatment planning systems partially due to the complexity in dose to BED conversion. To make real‐time BED‐base optimization possible, a fast dose–BED conversion must be established.

Use of multiple types of seeds and hybrid seeds in brachytherapy have been previously investigated.^7^, ^10^, ^12^ Hybrids seeds, equivalently called multi‐isotope seeds, are brachytherapy sources with more than one isotope. Hybrid seeds are typically denoted with a ratio in front to indicate the percentage of dose contribution from each isotope to a point in water that is one centimeter away from the center of the seed. For example, a 50/50 hybrid seed indicates 50% dose contribution from each isotope, while a 75/25 indicates 75% of the dose comes from the first isotope and 25% percent of the dose comes from the second isotope (at 1 cm in water). In this study, we used hybrid seeds that have a mixture of ${}^{125}I$ and ${}^{103}\text{Pd}$.

Depending on the dose delivery mechanism, calculation of BED from radiation dose is relatively straightforward. For fractionated external beam radiation therapy, BED follows:^(4)^

(1) $$BED=nd(1+\frac{d}{\alpha /\beta })‐\frac{\text{ln}2(T‐T_{k})}{\alpha \cdot T_{p}}$$

where, *n* is the number of fractions, *d* is the dose per fraction, *T* is the overall treatment time, *T* is the cell number doubling time, $T_{k}$ is time at which repopulation starts after treatment has started. The terms α and β are radiobiological parameters that categorize radiation effects on cells.

For brachytherapy using single isotope seeds, the BED equation can be approximated for clinical applications as:^(5)^

(2) $$BED=D\{1+2(d_{0}*\lambda )(\beta /\alpha )*(\frac{\kappa }{\mu ‐\lambda })\}‐\frac{.693*T}{\alpha \cdot T_{p}}$$

where

(3) $$\kappa =(\frac{1}{1‐e^{‐\lambda T_{eff}}})\{(\frac{1‐e^{‐2\lambda T_{eff}}}{2*\lambda })‐[\frac{1‐(e^{‐\lambda T_{eff}}*e^{‐\mu T_{eff}})}{\mu +\lambda }]\}$$

(4) $$T_{\mathrm{eff}}=\frac{1}{\lambda }*\text{ln}[(1.44*d_{0})*(\alpha *T_{p})]$$

Here, *D* is total dose, $d_{0}$ is the initial dose rate, μ is the repair coefficient, λ is the isotope decay constant, and $T_{\text{eff}}$ is the effective treatment time.

Due to the mixture of dose rates, the calculation of BED becomes more complicated for multi‐isotope seeds. The mixture of dose rate prevents an analytic expression of $T_{\text{eff}}$ and so a system of equations is needed for calculating BED. The equations necessary for calculating the BED for the multi‐isotope seeds are the BED equation^(7)^ and the time derivative of the BED equation.^(8)^

(5) $$\begin{array}{l} BED(t,d_{0i})=\sum_{i}^{N}\frac{d_{0i}}{\lambda_{i}}(1‐e^{‐\lambda_{i}t})+2(\frac{\beta }{\alpha })\sum_{i}^{N}(\frac{d_{0i}^{2}}{\mu ‐\lambda_{i}}\{\frac{1}{2\lambda_{i}}(1‐e^{‐2\lambda_{i}t})‐\frac{1}{\mu +\lambda_{i}}(1‐e^{‐(\mu +\lambda_{i})t})\}+\\ \frac{d_{0i}d_{0i+1}}{\mu ‐\lambda_{i}}\{\frac{1}{\lambda_{i}+\lambda_{i+1}}(1‐e^{‐(\lambda_{i}+\lambda_{i+1})t})‐\frac{1}{\mu +\lambda_{i+1}}(1‐e^{‐(\mu +\lambda_{i+1})t})\})‐\frac{0.693T_{eff}}{\alpha T_{p}} \end{array}$$

(6) $$\frac{\partial BED}{\partial t}{|}_{t‐Teff}=0$$

Here *N* is the number of different types of isotopes; N=2 for the 2‐isotope hybrid seeds.

Numerical computation of BED is possible; however, this process is time consuming.^(11)^ Without an efficient way of computing BED in hybrid brachytherapy, biological analysis of treatment plans becomes difficult. Thus, a fast dose–BED conversion method is desirable. The purpose of this paper is to introduce a fast dose–BED conversion so that BED‐based treatment plan optimization using hybrid brachytherapy seeds is feasible.

In Eq. (2), there is a unique correspondence between total dose and BED for a single isotope source. Therefore, a dose–BED conversion table can be easily established for single isotope seeds. However, for hybrid seeds it is possible that a total dose could have multiple corollary BED values due to the dependence of BED on the initial dose rates, which depend on the distance between the point of interest and the seed. Equation (7) is the formula for total dose, D, as calculated from the initial dose rate $d_{0i}$ of isotope i. Equation (8) shows the relation of initial dose rate $d_{0i}$ of isotope i with distance.

(7) $$D=\frac{T_{1/2i}}{\text{ln}(2)}d_{0i}$$

(8) $$d_{0i}=S_{i}\Lambda_{i}\sum_{j}^{N}\frac{gP_{i}(r_{j})\Phi_{an\_i}(r_{j})}{r_{j}^{2}}$$

where $r_{j}$ is distance, $S_{i}$ is the air kerma strength, $\Lambda_{i}$ is the dose rate constant, $gP_{i}(r_{j})$ is the radial dose function and $\Phi_{an\_\underset{˙}{i}}(r_{j})$ is the anisotropy function. It should be noted that these source specifications are construction‐dependent. The values of the dosimetric parameters are given in Table 1.

**Figure 1:**
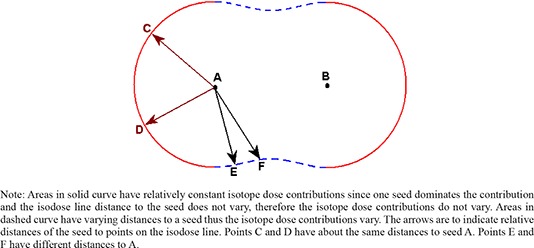
Illustration of an isodose line for total dose as a result of two hybrid seeds A and B. Total dose remains the same along the isodose line; however, individual isotope dose contributions vary.

Since each isotope has a different anisotropy and radial dose function, the ratio of dose contributions from the isotopes will vary with distance. This concept is illustrated in Fig. 1. Therefore, to generate a dose–BED conversion table for hybrid seeds, the relation of individual dose contributions to BED, (while maintaining a constant total dose) needs to be established.

**Table 1:** Values of dosimetric parameters for the hybrid sources.^(10)^ gP(r) and $\Phi_{\text{an}}(r)$ were linearly interpolated. Strengths, S, as well as the dose rate constant, Λ, are obtained from the manufacturer.^(10)^

*Isotope*	*S (50/50) (U)*	*S (75/25) (U)*	*S (25/75) (U)*	Λ *(cGy/h/U)*	*gP(r) (cm)*	$\Phi_{\text{an}}(r)\;(\text{cm})$	λ *(days)*
${}^{125}I$	0.22	0.33	0.11	0.95	1.111−0.139r	0.212+0.075r	59.4
${}^{103}\text{Pd}$	1.42	0.71	2.13	0.69	1.135−0.186r	0.267+0.012r	17

## II. MATERIALS AND METHODS

Because of the mixture of dose rates, isotope dose contributions along an isodose line will vary, influencing the BED. To see what variation this causes in BED vs. total dose graphs, the individual isotope dose components along an isodose line to the corresponding BED values needs to be examined. A hybrid seed distribution comprising of 49 seeds in a 512×512 voxel plane was generated. From this, the three‐dimensional (3D) dose distributions were calculated in a 512×512×3 grid with grid size of $0.65\times 0.65\times 3\;{\text{mm}}^{3}$ for each isotope using Eqs. (7) and (8). Using the dose distributions from each isotope, the BED distribution can be calculated using Eqs. (5) and (6). Prostate radiobiological parameters were assigned to each voxel. A graph of isotope dose contributions vs. BED can be made and analyzed to see how BED is influenced by the isotope dose contributions.

In the same region, varying the number of sources generates isodose lines along which isotope dose contributions vary. (A 50 Gy isodose line from a 2 seed distribution will have different dose contributions from each isotope as compared to a 50 Gy isodose lines from a 49 seed distribution.) This investigation examines the influence of the number of sources, as well as seed distribution on BED vs. the total dose using Eqs. (7) and (8). The 3D dose distributions were calculated for 2, 5, 10, and 49 seeds distributed in the volume. From these dose distributions, the BED distributions were calculated using Eqs. (5) and (6). Finally, the total dose values were coupled by voxel with their corresponding BED values, and a graph of dose component versus BED was constructed.

If the variation in BED values for a certain total dose was found to be large, then a dose–BED conversion table would not be useful, clinically. However, if the variation in BED is found to be small with respect to a total dose value, then practical applications of a dose–BED conversion table can be considered. A numerical approach was established to calculate BED distributions from initial dose rate distributions for hybrid seed implant brachytherapy. All numerical calculations were performed and coded for in‐house using Mathematica 7.0 software.

Next, a seed distribution and organ contours were taken from a postimplant CT image set in accordance with the TG‐43 protocol.^(13)^ The CT image set was a 512×512×24 voxel volume with voxel size $0.65\;\times \;0.65\;\times \;3\;{\text{mm}}^{3}$. This image set came from a patient previously treated with single isotope ${}^{125}I$ permanent seed implant brachytherapy. This plan was generated and contoured using the VariSeed program (Varian Medical Systems, Palo Alto, CA). The sources used in this plan were replaced with hybrid seeds. The seed strength was adjusted so that 110 Gy of BED, which corresponds to 145 Gy of dose for ${}^{125}I$ seeds, covered 90% of the prostate.^(14)^ A dose distribution was then calculated for this hybrid seed set. The BED distribution was calculated based on the dose distribution with the tissue types determined by the contours. Both the numerical method and the fast conversion method were applied to this case for the dose to BED conversion and compared. When the numerical approach was used, the component dose distributions were also used in the conversion, while the fast conversion only used the total dose distribution.

In addition to calculating dose–BED graphs for a 50/50 hybrid seed, other ratios were investigated, as well. Graphs were also generated for a 75/25 and 25/75 hybrid seed. Strengths of the seeds were adjusted to reflect the isotope ratios investigated. These strength values are given in Table 1. Note that $\Lambda_{i}$, ${\text{gP}}_{i}(r)$ and $\Phi_{\text{an}\_i}(r)$ remained the same for these ratios. Once the strengths were adjusted, dose distributions followed by BED distributions could be calculated.

Because BED can vary depending on the tissue medium, dose vs. BED figures were generated for multiple tissue types: prostate, bladder wall, rectum, and other normal tissue. Values for the α, β, and μ used in this study are given in Table 2. For each new set of biological parameters, the numerical method had to be applied to get the dose–BED correspondence data.

**Table 2:** Biological parameters used in dose–BED conversion for the various hybrid sources.^9^, ^14^, ^18^, ^23^

*Tissue*	α *(Gy* ^*‐1*^)	β *(Gy* ^*‐2*^)	μ *(days)*	$T_{p}$ *(days)*
Prostate Cancer	.15	0.048	0.013	40
Rectum	0.048	0.012	0.016	60
Bladder	0.077	0.02	0.031	60
Other Normal Tissue	0.2	0.067	0.049	60

For each total dose vs. BED graph, a line of best fit was determined using Excel's polynomial fit. Since these equations are analytic, fast calculations can be performed with them. In optimizing plans using hybrid seeds, these derived equations should be used.

## III. RESULTS

**Figure 2:**
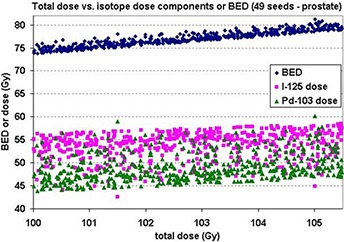
The individual isotope contributions to the total dose as well as BED to the total dose relation for 49 seeds is shown. This graph was calculated for the 50/50 hybrid seeds using the radiobiological parameters for prostate.

Because a hybrid source contains multiple isotopes, the dependency of individual isotope dose contributions to BED needs to be investigated. The BED, total dose, and individual dose components were calculated for a 49 seed distribution within a prostate medium for the 50/50 hybrid seed. The individual dose contributions for a given total dose leads to a BED variation seen in Fig. 2.

Figure 2 shows that for a specific total dose value, multiple individual dose contribution combinations from each isotope exist. This causes a variation in the BED value for a specific total dose. However, the variation in BED as compared to the variation in isotope dose contributions is smaller.

**Figure 3:**
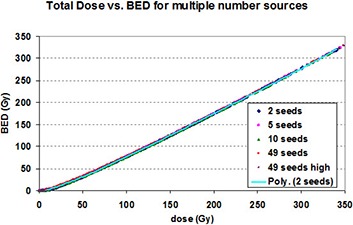
BED vs. the total dose for differing number of seeds and distributions is shown. This graph was calculated for prostate. “Poly” refers to the polynomial equation fitted to the indicated data.

Next, an investigation as to whether the number of seeds influences the BED value was investigated. Figure 3 shows the total dose to BED conversions for a larger range of values for the 50/50 hybrid seed. The data points for 2, 5, 10, and 49 seeds are seen to overlap, suggesting that number of seeds does not influence the relation of total dose to BED.

**Figure 4:**
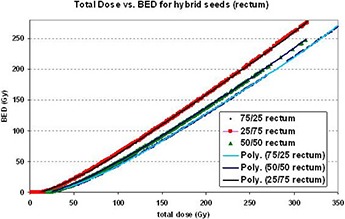
BED vs. the total dose calculated for the rectum for hybrid seeds of ratios 25/75, 50/50, and 75/25 are shown. “Poly” refers to the polynomial equation fitted to the indicated data.

Similar BED uncertainty due to individual dose contribution variations was found for 75/25 and 25/75 hybrid seeds. Since the BED variation was seen to be small, generating total dose vs. BED graphs can be done. Figure 4 shows the total dose vs. BED graphs for rectal tissue for hybrid seed ratios of: 50/50, 75/25, and 25/75. From these graphs, polynomial equations can be fitted. These equations allow for a quick conversion of total dose to BED. For other tissues (prostate, bladder, and other normal tissue), the numerical technique was applied in order to obtain the total dose vs. BED dataset. A polynomial equation was generated to fit each dataset. For these tissues types, the three isotope ratios were investigated.

A polynomial equation to the fourth power was generated by Excel for the following scenarios: rectal, prostate, bladder, and other normal tissues for hybrid seeds of ratio: 50/50, 25/75, and 75/25. Coefficients for these equations, as well as the maximum differences between the BED values calculated using the fitted equations and the ones from direct conversion, are listed in Table 3. The $R^{2}$ values for all the fits are >0.999. The general form of the polynomial fit is given in Eq. (9).

(9) $$BED(D)=AD^{4}+BD^{3}+CD^{2}+ED+F$$

**Table 3:** Coefficients of the fits for the various conversion curves and the maximum differences between the fitted curves and the direct conversions.

				*Coefficients*			
*Hybrid Seed Type*	*Organ*	*A*	*B*	*C*	*E*	*F*	*Max Error (Gy)*
50/50	Prostate	$1.074\times {\mathrm{10}}^{-8}$	$-8.877\times {\mathrm{10}}^{-6}$	$2.802\times {\mathrm{10}}^{-3}$	0.605	−4.765	1.03
50/50	Rectum	$1.670\times {\mathrm{10}}^{-8}$	$-1.372\times {\mathrm{10}}^{-5}$	$4.489\times {\mathrm{10}}^{-3}$	0.246	−7.792	1.75
50/50	Bladder	$3.960\times {\mathrm{10}}^{-7}$	$-1.216\times {\mathrm{10}}^{-4}$	$1.520\times {\mathrm{10}}^{-2}$	−0.038	−2.192	1.65
50/50	NT	$9.729\times {\mathrm{10}}^{-9}$	$-8.023\times {\mathrm{10}}^{-6}$	$2.968\times {\mathrm{10}}^{-3}$	0.661	−4.923	1.53
75/25	Prostate	$2.000\times {\mathrm{10}}^{-8}$	$-1.000\times {\mathrm{10}}^{-5}$	0.004	0.465	−4.160	0.85
75/25	Rectum	$2.000\times {\mathrm{10}}^{-8}$	$-2.000\times {\mathrm{10}}^{-5}$	0.006	0.015	−1.979	1.02
75/25	Bladder	$1.917\times {\mathrm{10}}^{-8}$	$-1.643\times {\mathrm{10}}^{-5}$	$5.360\times {\mathrm{10}}^{-3}$	0.205	−3.784	1.26
75/25	NT	$1.171\times {\mathrm{10}}^{-8}$	$-9.750\times {\mathrm{10}}^{-6}$	$3.325\times {\mathrm{10}}^{-3}$	0.594	−5.261	1.13
25/75	Prostate	$9.056\times {\mathrm{10}}^{-9}$	$-7.186\times {\mathrm{10}}^{-6}$	$2.232\times {\mathrm{10}}^{-3}$	0.708	−4.388	0.96
25/75	Rectum	$2.222\times {\mathrm{10}}^{-8}$	$-1.713\times {\mathrm{10}}^{-5}$	$5.017\times {\mathrm{10}}^{-3}$	0.321	−4.402	1.08
25/75	Bladder	$1.418\times {\mathrm{10}}^{-8}$	$-1.714\times {\mathrm{10}}^{-5}$	$3.868\times {\mathrm{10}}^{-3}$	0.494	−5.209	1.32
25/75	NT^a^	$7.586\times {\mathrm{10}}^{-9}$	$-6.170\times {\mathrm{10}}^{-6}$	$2.567\times {\mathrm{10}}^{-3}$	0.744	−4.682	1.50

^a^ NT=normal tissue

**Figure 5:**
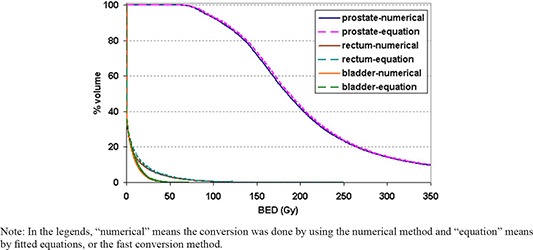
Comparison of BED–volume histograms between the BED values converted directly from the numerical approach and the ones calculated using the fitted equations for a clinical treatment plan with the ${}^{125}I$ seeds replaced by the 50/50 hybrid seeds and seed strength adjusted.

Figure 5 shows a comparison of the BED–volume histogram (BEDVH) based on the treatment plan with ${}^{125}I$ seeds replaced by the 50/50 hybrid seeds. The comparison is between the BED values converted from dose using numerical approach and the ones calculated with the fitted equations. The curves by the equations closely match the ones converted directly by the numerical approach. While the numerical approach took over 4 hours to convert the total dose to BED, the fast conversion method did the conversion for the whole volume of various tissue types for this plan in about 1 second on a 2.66 GHz PC.

## IV. DISCUSSION

The reason for obtaining data from a 2, 5, 10, and 49 seed distributions was to analyze local seed influence compared to an overall distribution. Seed placement in brachytherapy plans may sometimes involve placing seeds that are relatively far from other seeds. Therefore, it needs to be determined if local seed influence differs from the overall distribution when generating the total dose vs. BED graphs. If it was seen that the total dose vs. BED graph for a 2 seed distribution (local influence) was different then a total dose vs. BED graph from a 49 seed distribution, then these generated graphs would not be useful.

For specific total dose values, a variation in BED values was seen. This is due to BED being a function of individual isotope dose contributions and not only the total dose. Since a number of possible combinations exist to give rise to a specific total dose, this was to be expected. However, the variation in BED was considered small. (The maximum variation in BED value was seen to be ~2 Gy. When BED is 100 Gy, it is an error of 2%.) An explanation for this is that not all possible combinations of isotope dose contributions that add up to a specific total dose exist in the total isodose lines due to the limit of their radial dose and anisotropy functions.

This fast conversion of total dose to BED for low total dose values is not as accurate as it is for high dose values. The variation in BED values for total dose values around 30 Gy is roughly the same as in higher total dose values (~2 Gy). This results in a relative uncertainty of ~7%. Therefore, the relative uncertainty for low BED values when compared to large BED is greater (even though the absolute variation was seen to be the same).

The comparison of the BED–volume histograms between the directly converted BED values by the numerical method and the ones by the fitted equations for a treatment plan shows the difference is minimal, as shown in Fig. 5. This indicates that this fast conversion method can be applied clinically with acceptable tolerance. The simplicity of the fast conversion method is reflected by the conversion time difference between the two methods. The short‐time conversion is the major advantage of this method, which makes the BED‐based plan optimization possible in brachytherapy planning.

The update for TG‐43^(13)^ recommends a 5th order polynomial be used as the equation of fit for the anisotropy and radial dose functions. Due to calculation time, a first order approximation was used for the anisotropy and radial dose functions. The applicable range for the radial dose and anisotropy functions listed is 8 cm. For a more accurate dose distribution beyond this feasibility study, other types of functions should be used for the fittings.^15^, ^16^

This method can also be confidently applied to single isotope seeds, in which the dose to BED conversion would be a one‐to‐one correspondence (the 2 Gy uncertainty of BED would not exist), which makes the conversion more accurate.

TG‐137 states nominal values for α/β ratios when performing BED calculations for the prostate.^(17)^ Within this study, an α/β ratio of 3.1 was used (TG‐137 states to use an α/β ratio of 3.0). A value of 40 days was assigned to $T_{p}$ (TG‐137 states to use a $T_{p}$ of 42 days). It was found that difference in using the TG‐137 values amounted to a .3% decrease in calculating the BED. If a set of biological parameters of great differences is used, a new dose–BED conversion curve needs to be calculated.

When using the fitted equation to convert dose to BED, for very low doses, the BED values may be negative, which has no physical meaning. Whenever BED<0, it should be taken as BED=0.

## V. CONCLUSIONS

The work presented in this paper provides a fast calculation method of converting total dose to BED for 50/50, 75/25, and 25/75 isotope ratios within hybrid seeds. These conversions are calculated for specific organs: prostate, bladder, rectum, and other normal tissue. In providing a fast calculation method, the need for solving a series of transcendental equations was bypassed. This allows for BED‐based optimization of treatment plans using these novel brachytherapy sources. This method can also be applied to other single isotope sources with confidence.

## Supporting information

Supplementary Material Files
