# Supplementary figures and images for: Providing a fast conversion of total dose to biological effective dose (BED) for hybrid seed brachytherapy
Source: J Appl Clin Med Phys. 2012 Sep 6;13(5):24–32. doi: 10.1120/jacmp.v13i5.3800 (PMC5718217; doi:10.1120/jacmp.v13i5.3800)

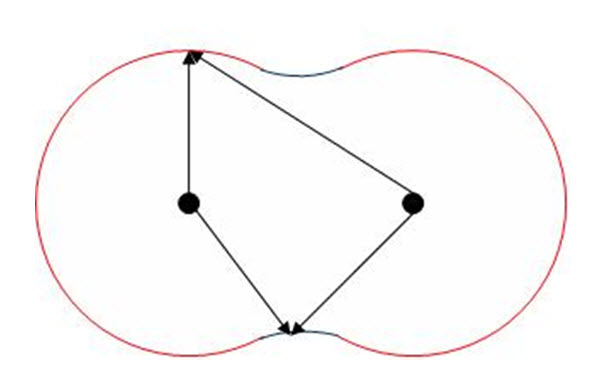

Supplement: Supplementary file 1 — Supplementary Material Files [file ACM2-13-024-s001.jpg]
